# Comprehensive 3D analysis of condylar morphology in adults with different skeletal patterns – a cross-sectional study

**DOI:** 10.1186/s13005-020-00245-z

**Published:** 2020-11-30

**Authors:** Petra Santander, Anja Quast, Carolin Olbrisch, Marius Rose, Norman Moser, Henning Schliephake, Philipp Meyer-Marcotty

**Affiliations:** 1 Department of Orthodontics, University Medical Center Goettingen, Robert-Koch-Str. 40, 37075 Goettingen, Germany; 2 Department of Oral and Maxillofacial Surgery, University Medical Center Goettingen, Robert-Koch-Str. 40, 37075 Goettingen, Germany

**Keywords:** Temporomandibular joint, Skeletal class, Vertical relationship, Asymmetry, Condylar volume, Shape, Inclination, CBCT

## Abstract

**Background:**

The correlation between individuals’ condylar morphology and their skeletal pattern is of great interest for treatment strategies ranging from orthodontic orthopaedics to orthognathic surgery. The objective of the present study was to investigate this relationship three-dimensionally.

**Methods:**

A total of 111 adult patients (mean age = 27.0 ± 10.2 years) who underwent head computed tomography or cone beam computed tomography scans were included. Based on these data, 3D models of the skull and the condyles were calculated. The craniofacial skeleton was evaluated (1) transversally regarding skeletal symmetry (menton deviation), (2) sagittally regarding skeletal classes (Wits appraisal) and vertically regarding the inclination of the jaws (maxillomandibular plane angle). The condylar morphology was assessed (a) linearly by the condylar width, height and depth; (b) angularly by the antero-posterior and medio-lateral condylar inclination; and (c) volumetrically by the ratio of the condylar volume/mandibular volume (C/Mand).

**Results:**

(1) Transversal: Asymmetric patients showed significantly higher discrepancies in the volumetric ratio C/Mand on the deviation and non-deviation side compared to symmetric patients. (2) Sagittal: Class III subjects demonstrated longer, more voluminous condyles with higher antero-posterior and medio-lateral inclination angles compared to Class II participants. (3) Vertical: Hyperdivergent subjects had smaller condyles with higher antero-posterior inclination angles than those of hypodivergent subjects. No interactions of skeletal class and vertical relationships regarding condylar morphology were observed.

**Conclusions:**

This study demonstrates a clear correlation between pronounced skeletal patterns and condylar morphology in an adult population. The description of radiographic condyle characteristics in relation to the craniofacial morphology improves orthodontic treatment planning and could be helpful in the diagnosis of temporomandibular joint pathologies.

## Background

The condyles are very adaptable and the most important growth sites in the craniofacial complex [1]. Their cartilaginous tissue contributes to the capability of remodelling in response to external stimuli, even after natural growth [2]. Therefore, the condyles are not only the major target in orofacial orthopaedics [3, 4], but are considered to determine long-term stability in orthognathic surgery [5, 6].

As form and function are closely linked, correlations between condylar characteristics and craniofacial morphology are expected by most orthodontists. In his longitudinal implant study, Björk described mandibular growth from child- to adulthood [1, 7, 8]. He postulated several structural characteristics of the mandible to predict the direction of growth. Due to major variances in the condylar growth rate, only one morphological feature was identified in the condyle: its inclination.

The adaptability and versatility of the condyle complicate the discrimination whether an observed morphology is an anatomical variance, a response to functional adaptation or a pathological sign. Systematic assessment enhances the clarity of condylar pathophysiology and enables us to identify radiological abnormalities such as flattening, osteophytes, erosions, and cysts [9]. However, systematic analyses of physiological condyles are rare, and the correlations between the craniofacial skeleton and the condyles often just indicate tendencies [10, 11].

With today’s widely available three-dimensional (3D) data from computer tomography (CT) and cone beam computed tomography (CBCT), some efforts have been undertaken to gain more knowledge about condylar morphology [10–16]. Tecco et al. [16] investigated gender effects on condylar surface and volume without considering any skeletal aspects. Other authors examined the correlation of condylar characteristics and skeletal patterns, but only in the sagittal and vertical dimensions [10, 15]. However, the transversal dimension has to be taken into account since asymmetric patients demonstrate higher variations in condylar characteristics between both condyles than symmetric patients [14].

Therefore, the objective of the present study was to systematically investigate condylar characteristics by 3D volumetric, linear and angular measurements in transversal, sagittal, and vertical dimensions in an adult population. We hypothesized, that there is:

1. a difference in condylar characteristics between the deviated and non-deviated side in asymmetric patients,
2. a correlation between condylar characteristics, especially condylar volume, and skeletal classes, and
3. a correlation between condylar characteristics, especially condylar inclination and vertical relationship.

## Methods

In accordance with the declaration of Helsinki, this study was approved by the institutional ethics committee (application number 7/1/16). All patients gave their written informed consent to participate in the study. The manuscript was prepared in conformance with the STROBE Statement [17].

### Subjects

This cross-sectional study included 111 adult patients who underwent head CT or CBCT scans at the Department of Orthodontics and/or the Department of Oral and Maxillofacial Surgery at our institution between 2016 and 2019. The indication for the CT/CBCT scan was one of the following: orthognathic surgery due to severe malocclusion, suspected midface fractures, traumatic brain injury or inflammatory processes. Inclusion criteria were the availability of a complete CT/CBCT scan of the viscerocranium and the anterior cranial base, the patient’s consent to participate in the study and symmetric bilateral occlusal support in the molar area. Exclusion criteria were cleft lip and palate, mandibular fractures, craniofacial anomalies, head and neck oncological diseases and abnormal condylar morphology due to the development of temporomandibular osteoarthritis, such as erosion, sclerosis, or osteophytes.

For sample size determination, effect size was estimated according to a previous assessment of the right condylar volume in normo-, hypo- and hyperdivergent patients (10). Calculation in G*Power (v. 3.1.9.2, University of Düsseldorf) [18] revealed a sample size of 111 participants (MANOVA: Global effects; α = 0.05; 1-β = 0.95; *f*^*2*^ *=* 0.1225). Eligible patients were included consecutively without further selection and without consideration of their skeletal patterns. In total, 183 patients were screened, and 72 patients were rejected by the study protocol.

### Methods

For all participants, the skull and jaw “Digital Imaging and Communications in Medicine (DICOM)” data from CT (Spiral Scan, Somatom Definition AS, Siemens Healthcare GmbH, Erlangen, Germany; 128-line multi-detector, layer thickness 0.6 mm) or CBCT scans (PaX Zenith 3D, OrangeDental, Biberach an der Riss, Germany; field of view 240 × 190 mm, voxel size 0.3 mm) were exported for the creation of a 3D model.

**Fig. 1:**
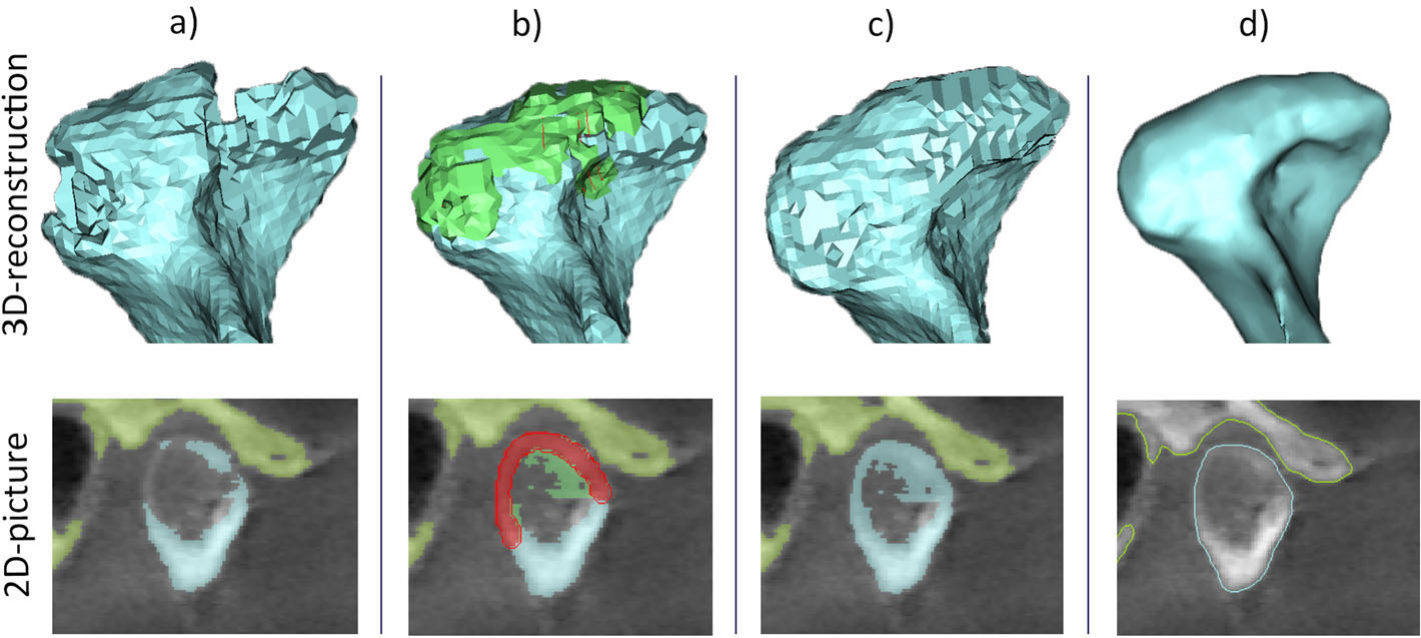
Manual processing of the condyle by adapting the threshold and smoothing the surface. **a** Initial three dimensional image from the “digital imaging and communications in medicine” (DICOM) data; **b** manual correction of the threshold, layer by layer, resulting in image (**c**); **d** automatic smoothing of the surface

The processing of the DICOM data started using the software “Mimics inPrint” (Materialise, Leuven, Belgium). Bone tissue was segmented by setting the threshold range individually with visual control of the selected region. For segmentation of the condyle and the fossa articularis, the threshold was set between − 89 and 689 units. The maximum threshold was 2047 units. The soft tissue-like grayscale levels and low contrast in the temporomandibular region required a further individual adjustment of the threshold’s lower border and manual processing, layer by layer, in all three dimensions to achieve accurate bone representation (Fig. 1).

Next, a 3D model of the skull and mandible was automatically calculated and smoothed. For cephalometric analysis, the 3D model was aligned to the Frankfurt Horizontal (FH) plane and exported.

### Measurements

**Fig. 2:**
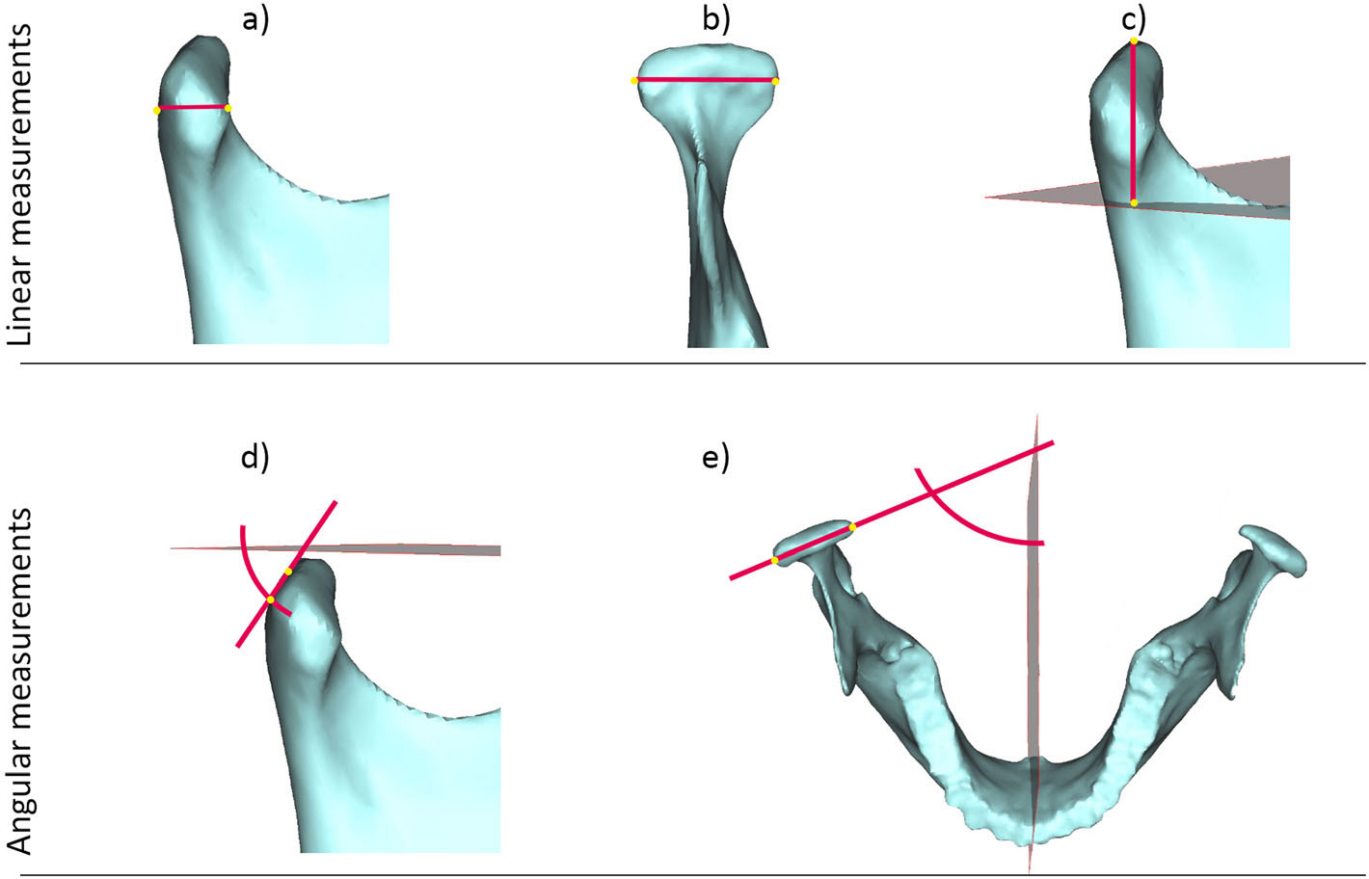
Measurements on the three dimensional model of the condyle and mandible. Linear measurements: **a** condylar depth; **b** condylar width; and **c** condylar height. Angular measurements: **d** antero-posterior inclination of the condyle; and **e** Medio-lateral inclination of the condyle

**Fig. 3:**
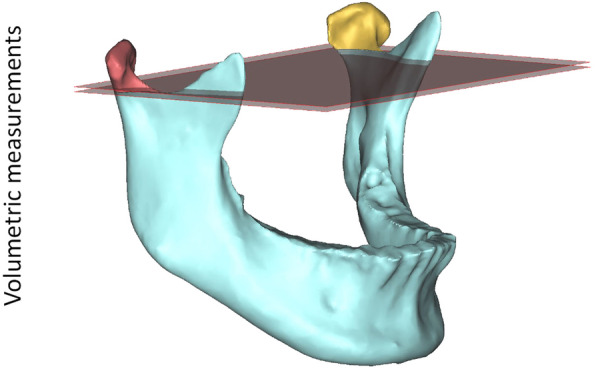
Volumetric measurements of the right and left condyle. The condylar volume was limited by a plane parallel to the Frankfurt Horizontal plane through the most caudal point of the mandibular notch (teeth were removed). The plane was constructed for each side separately. In asymmetric patients, the calculated ratio of the condylar volume/mandibular volume was significantly higher on the non-deviation side (yellow) compared to the deviation side (red)

The linear, angular and volumetric measurements were performed by one single examiner (C.O.) on the 3D model in ProPlan CMF (Materialise, Leuven, Belgium). Each condylar measurement was performed on the right and left sides separately and was checked for correctness in the sagittal, axial and coronal slices of the CT/CBCT. All measurements are listed in Table 1 and illustrated in Figs. 2 and 3. The antero-posterior inclination angle of the condyle was projected on the midsagittal plane and the lateral inclination angle of the condyle on the FH plane. For measurement of the mandibular volume, including the alveolar process, the teeth were removed at the level of the alveolar margin. To assess intrarater agreement, the same examiner indicated the landmarks of 10 randomly selected subjects on a second occasion at least 6 months later. For interrater agreement, a second examiner performed the measurements on 10 3D models.

**Table 1:** Definition of linear, angular and volumetric measurements

Measurement	Abbreviation	Definition
*Linear*		
Condylar depth	C depth	Shortest distance between the most posterior and the most anterior point of the condylar head
Condylar width	C width	Shortest distance between the most lateral and the most medial point of the condylar head
Condylar height	C height	Shortest distance between the most cranial point of the condylar head and a plane parallel to FH through the most caudal point of the mandibular notch
*Angular*		
Antero-posterior inclination of the condyle	C incl a-p	Angle between the FH plane and the connecting line between the most cranial and the most posterior point of the condylar head
Medio-lateral inclination of the condyle	C incl m-l	Angle between the midsagittal plane and the connecting line between the most medial and the most lateral point of the condylar head
*Volumetric*		
Condyle	C	Condylar volume limited by a plane parallel to FH through the most caudal point of the mandibular notch
Mandible	Mand	Total mandibular volume including the alveolar process (without teeth)
Ratio C/Mand	C/Mand	$\frac{condylar\ volume}{mandibular\ volume}$ × 100

### Statistical analysis

Statistical analysis was performed using SPSS v.22 (IBM, New York). Normal distribution was confirmed by q-q-plots. The sample was categorized according to the transversal, sagittal and vertical relation. To determine the degree of asymmetry (transversal dimension), the deviation of menton (Me) from the midsagittal plane (MSP) was used [19]. The sample was classified as symmetric (ME-MSP < 2 mm), moderately asymmetric (ME-MSP 2–4 mm) and strongly asymmetric (ME-MSP > 4 mm).

To define the skeletal class, the Wits appraisal [20] was used. This method uses perpendiculars from the points A and B onto the occlusal plane. The group was categorized in skeletal Class I (Wits − 2 to 2 mm), Class II (Wits > 2 mm) and Class III (Wits < − 2 mm).

The vertical relationship was defined by the maxillomandibular plane angle. This angle is defined by the Mandibular-Line (ML) (determined by Gonion to Menton) and the Nasal-Line (determined by Anterior-Nasal-Spine to Posterior-Nasal-Spine) [21]. The sample was classified as neutral (ML-NL = 20.5 ° to 26.5°), hypodivergent (ML-NL < 20.5°) and hyperdivergent (ML-NL > 26.5°).

Unequal sample sizes were balanced using bootstrapping (1000 bootstrap samples, BCa intervals) and the mean, the standard deviations and the bootstrapped 95% confidence intervals (95% CI) of the mean were reported. Intra- and interrater agreements of all measurements were assessed by Bland-Altman-plots [22]. Age and gender were regarded as potential confounders. They were compared between the transversal, sagittal and vertical skeletal patterns by univariate analysis of variance.

For the evaluation of possible side differences associated with the degree of asymmetry, means (*M*) and standard deviations (*SD*) of the difference between the morphological characteristics on the deviation and the non-deviation side were determined. To calculate this difference the measurement on the side from which menton deviated was subtracted from the side to which menton deviated. The interaction between these differences and the degree of asymmetry were assessed by a multivariate analysis of variance (MANOVA). α-level was set at 0.05. Multicollinearity was excluded by Pearson correlation. The homogeneity assumption was proved by Box’s test. F- and *p*-values were reported according to Pillai’s trace.

For skeletal class and vertical relationship, the measurements of both sides were averaged per patient. For the ratio C/Mand, both sides were added. The effects of sagittal and vertical skeletal patterns on condylar morphology characteristics were evaluated using a MANOVA.

The MANOVAs were followed up with stepwise discriminant analysis to determine which morphological characteristics discriminated best the skeletal patterns.

## Results

In total, we investigated the morphological characteristics of 222 condyles in 111 participants (for a detailed description of the study sample, see Table 2). Bland-Altman plots revealed high intra- and interrater agreements for linear and volumetric measurements (Table 3). For angular measurements, interrater agreement was less good, but still within clinical acceptable limits.

**Table 2:** Demographic and clinical characteristics of the participants

Participants	*N* = 111
Male	*n* = 49
Female	*n* = 62
Participants’ age	*M* = 27.0 years; *SD* = 10.2 years
Participants by asymmetry	
Symmetry (Me-MSP < 2 mm)	*n = 65* (*M* = 0.8 mm; *SD* = 0.6 mm)
Moderate asymmetry (Me-MSP = [2; 4 mm])	*n* = 22 (*M* = 2.9 mm; *SD* = 0.6 mm)
Strong asymmetry (Me-MSP > 4 mm)	*n = 24* (*M* = 6.2 mm; *SD* = 1.9 mm)
Participants by skeletal class	
Skeletal Class I (Wits = [− 2; 2 mm])	*n = 25* (*M* = − 0.8 mm; *SD* = 1.2 mm)
Skeletal Class II (Wits > 2 mm)	*n* = 36 (*M* = 6.5 mm; *SD* = 2.9 mm)
Skeletal Class III (Wits < − 2 mm)	*n =* 50 (*M* = − 9.1 mm; *SD* = 4.3 mm)
Participants by vertical relation	
Neutral (ML-NL = [20.5; 26.5°])	*n* = 32 (*M* = 23.2°; *SD* = 1.8°)
Hypodivergent (ML-NL < 20.5°)	*n* = 37 (*M* = 16.3°; *SD* = 4.1°)
Hyperdivergent (ML-NL > 26.5°)	*n* = 42 (*M* = 32.7°; *SD* = 4.3°)
Investigated condyles	*N* = 222

*ML-NL* Maxillomandibular plane angle, *Me-MSP* Menton deviation from the midsagittal plane

**Table 3:** Assessment of intra- and interrater agreement by Bland-Altman-plots revealed reliable and clinical acceptable measurements

Measurement	Agreement	*M*_Diff_	*SD*	LoA+	LoA-
C depth [mm]	Intra	−0.1	0.4	0.8	−0.9
	Inter	−0.5	0.8	1.1	−2.0
C width [mm]	Intra	0.5	0.5	1.5	−0.6
	Inter	0.7	0.5	1.7	−0.3
C height [mm]	Intra	−0.2	0.8	1.4	−1.9
	Inter	−0.5	1.0	1.5	−2.5
C incl a-p [°]	Intra	−0.1	3.8	7.3	−7.5
	Inter	4.4	9.7	23.3	−14.6
C incl m-l [°]	Intra	0	1.4	2.6	−2.7
	Inter	−2.2	3.0	3.7	−8.0
Ratio C/Mand. volume [%]	Intra	0	0	0	0
	Inter	0	0.1	0.1	−0.1

*a-p* Antero-posterior, *m-l* Medial-lateral, *c* Condylar, *mand* Mandibula

The sample was equally distributed according to age and gender between the vertical and symmetry groups (*p* > .05). For skeletal classes, no effect of gender was observed, while participants with skeletal Class III were significantly younger compared to patients with skeletal Class I or II (*p* = .001).

Using menton deviation from the midsagittal plane, the study sample was categorized as symmetric, moderately asymmetric and strongly asymmetric participants, and differences in condylar morphology between the deviation and non-deviation sides were assessed (Table 4). MANOVA indicated a significant effect of the degree of asymmetry and side-specific differences in condylar morphology, *F* (12, 208) = 2.18, *p* = .014, η^2^ = .11.

**Table 4:** Means (*M*), standard deviations (*SD*) and 95% confidence intervals of the mean (95% CI) of condylar morphology characteristics according to the degree of asymmetry

	Symmetry (*n* = 65)		Moderate asymmetry (*n* = 22)		Strong asymmetry (*n* = 24)	
	*M (SD)*	*95% CI*	*M (SD)*	*95% CI*	*M (SD)*	*95% CI*
Diff C depth [mm]	−0.1 (1.2)	[−0.4–0.2]	0.1 (1.0)	[− 0.3–0.5]	0.4 (1.1)	[0–0.9]
Diff C width [mm]	0.1 (1.4)	[−0.3–0.4]	0.7 (1.6)	[0–1.3]	0.6 (2.1)	[−0.3–1.4]
Diff C height [mm]	0.1 (1.3)	[−0.2–0.4]	0.8 (1.4)	[0.2–1.4]	0.8 (1.5)	[0.2–1.4]
Diff C incl a-p [°]	2.3 (8.8)	[0.2–4.5]	1.3 (7.7)	[−1.8–4.6]	1.0 (9.7)	[−2.7–4.7]
Diff C incl m-l [°]	−0.4 (7.8)	[− 2.4–1.4]	−1.7 (10.1)	[−6.2–2.0]	−1.0 (10.1)	[−5.4–2.9]
Diff Ratio C/Mand volume [%]	0 (0.4)	[−0.1−0.1]	0.4 (0.3)	[0.2−0.5]	0.5 (0.5)	[0.3−0.7]

*a-p* Antero-posterior, *m-l* Medial-lateral, *c* Condylar; *mand* Mandibula

Discriminant analysis showed that the ratio C/mand accounted for 100% of variance, canonical *R*^*2*^ = .2. All other variables were removed from the model. The ratio C/mand significantly discriminated between the transversal groups, Λ = 0.8, χ^2^ = 24.4, *p* < .001. Using this function, 63.1% of the cases were classified correctly into their transversal groups (Table 5).

**Table 5:** Classification of the symmetry group using the ratio C/mand

Original group	Predicted group			
	Symmetry	Moderate Asymmetry	Strong asymmetry	In total
Symmetry: n (%)	61 (93.8)	0 (0)	4 (6.2)	65
Moderate asymmetry: n (%)	16 (72.2)	0 (0)	6 (27.3)	22
Strong asymmetry: n (%)	15 (62.5)	0 (0)	9 (37.5)	24

For subsequent analysis, the condylar morphology characteristics of both sides were averaged per patient. The Wits appraisal divided the participants into skeletal classes I, II and III. The condylar morphology of participants with skeletal Class III differed noticeably from participants with skeletal Class I or II (Table 6). Participants with skeletal Class III demonstrated smaller condylar depth compared to Class I participants. They also showed higher angles of antero-posterior and medio-lateral condylar inclination compared to Class II participants. The ratio C/Mand and condylar height were higher in Class III than in Class I or II cases.

**Table 6:** Means (*M*), standard deviations (*SD*) and 95% confidence intervals of the mean (95% CI) of condylar morphology characteristics according to skeletal classes

	Class I (*n* = 25)		Class II (*n* = 36)		Class III (*n* = 50)	
	*M (SD)*	*95% CI*	*M (SD)*	*95% CI*	*M (SD)*	*95% CI*
C depth [mm]	8.5 (1.1)	[8.1–9.0]	8.3 (1.5)	[7.8–8.8]	7.5 (1.4)	[7.1–7.9]
C width [mm]	19.8 (2.2)	[19.0–20.6]	19.2 (2.8)	[18.3–20.1]	20.2 (2.5)	[19.5–21.0]
C height [mm]	16.7 (1.8)	[16.0–17.4]	16.6 (2.8)	[15.8–17.6]	18.5 (2.9)	[17.6–19.2]
C incl a-p [°]	56.9 (6.8)	[54.3–59.4]	53.9 (8.4)	[51.3–56.7]	60.9 (7.4)	[58.9–62.9]
C incl m-l [°]	67.5 (9.6)	[63.8–71.3]	64.2 (9.8)	[61.1–67.6]	69.9 (7.5)	[67.8–72.1]
Ratio C/Mand. volume [%]	5.8 (1.1)	[5.4–6.2]	5.6 (1.2)	[5.2–6.1]	6.6 (1.3)	[6.3–7.0]

*a-p* Antero-posterior, *m-l* Medial-lateral, *c* Condylar, *mand* Mandibula

The maxillomandibular plane angle categorized participants as hypodivergent, neutral and hyperdivergent. No differences in condylar morphology were observed between neutral and hypodivergent or neutral and hyperdivergent participants (Table 7). However, condylar characteristics of hypo- and hyperdivergent cases showed differences. Hyperdivergent cases had a smaller condylar depth, a smaller condylar width and a higher angle of antero-posterior inclination compared to hypodivergent cases.

**Table 7:** Means (*M*) and standard deviations (*SD*) of condylar (c) morphology characteristics and mandibular (mand) volume according to vertical skeletal patterns

	hypodivergent (*n* = 37)		neutral (*n* = 32)		hyperdivergent (*n* = 42)	
	*M (SD)*	*95% CI*	*M (SD)*	*95% CI*	*M (SD)*	*95% CI*
C depth [mm]	8.5 (1.6)	[8.0–8.9]	8.0 (1.4)	[7.4–8.4]	7.6 (1.1)	[7.3–8.0]
C width [mm]	20.5 (2.4)	[19.7–21.2]	20.3 (2.3)	[19.4–21.1]	18.9 (2.6)	[18.1–19.7]
C height [mm]	17.8 (2.6)	[17.0–18.6]	17.1 (3.1)	[16.1–18.2]	17.4 (2.7)	[16.7–18.2]
C incl a-p [°]	55.0 (7.9)	[52.4–57.4]	58.3 (8.8)	[55.4–61.5]	59.6 (7.4)	[57.4–62.1]
C incl m-l [°]	68.7 (9.8)	[65.7–71.9]	68.5 (7.2)	[65.9–71.1]	65.6 (9.5)	[62.8–68.6]
Ratio C/Mand. volume [%]	6.4 (1.2)	[6.0–6.8]	6.3 (1.3)	[5.9–6.8]	5.7 (1.3)	[5.3–6.1]

*a-p* Antero-posterior, *m-l* Medial-lateral, *c* Condylar, *mand* Mandibula

MANOVA indicated significant effects of sagittal, *F* (12, 196) = 4.56, *p* < .001, η^2^ = .22, and vertical, *F* (12, 196) = 1.84, *p* = .044, η^2^ = .1, skeletal patterns on condylar morphology with large and medium effect sizes, respectively, but no interaction effect of both, *F* (24, 400) = 0.5, *p* = .979. The MANOVA was followed up with stepwise discriminant analysis for sagittal and vertical relationships to develop a model that classifies group membership.

Discriminant analysis for skeletal class revealed two discriminant functions, which removed condylar width and condylar height from the model. Condylar depth, the ratio C/Mand, and antero-posterior and medio-lateral inclinations were included in both functions. The first function explained 89% of the variance, canonical *R*^*2*^ = .37, whereas the second explained only 11%, canonical *R*^*2*^ = .07. In combination, these functions significantly discriminated the skeletal classes, Λ = 0.59, χ^2^ = 56.6, *p* < .001. Removing the first function, the second function did not differentiate the sagittal groups significantly, Λ = 0.93, χ^2^ = 7.5, *p* = .058.

The correlations between outcomes and the discriminant functions indicated that the ratio C/mand and the antero-posterior and medio-lateral condylar inclinations loaded more highly on the first function (r_ratio C/Mand volume_ = .49; r_c incl a-*p*_ = .53; r_c incl m-l_ = .36) than on the second function (r_ratio C/Mand volume_ = −.14; r_c incl a-*p*_ = .22; r_c incl m-l_ = .29), whereas condylar depth loaded more highly on the second function (r_c depth_ = .53) than on the first function (r_c depth_ = −.41). The discriminant function plot revealed that the first function differentiated skeletal Class III from skeletal Class II, while the second function discriminated skeletal Class I from skeletal Class II and III. Using both functions, 65.8% of the investigated cases were correctly classified in their sagittal group. In particular, participants with skeletal classes II and III were clearly identified. The model rated them properly in 72.2 and 80% of cases, respectively. However, condylar morphology characteristics were insufficient to classify skeletal Class I, as 72% of Class I cases were falsely identified as Class II or III cases (Table 8).

**Table 8:** Classification of the skeletal class using the ratio C/mand, the antero-posterior and medio-lateral condylar inclination and the condylar depth

Original group	Predicted group			
	Class I	Class II	Class III	In total
Class I: n (%)	7 (28.0)	11 (44.0)	7 (28.0)	25 (100)
Class II: n (%)	3 (8.3)	26 (72.2)	7 (19.4)	36 (100)
Class III: n (%)	5 (10.0)	5 (10.0)	40 (80.0)	50 (100)

The pair of functions in the discriminant analysis for the vertical relationship included condylar width and antero-posterior condylar inclination. Condylar height, depth, medio-lateral inclination and the ratio C/Mand volume were excluded from the model. The first function explained 94.2% of the variance, canonical *R*^*2*^ = .15, whereas the second explained only 5.8%, canonical *R*^*2*^ = .01. In combination, these functions significantly differentiated the vertical groups, Λ = 0.84, χ^2^ = 18.3, *p* = .001, but removing the first function revealed that the second function did not significantly discriminate the vertical groups, Λ = 0.99, χ^2^ = 1.1, *p* = .288. Condylar width loaded highly onto both functions (r_1_ = .69; r_2_ = .72), while antero-posterior condylar inclination loaded more highly on the second function (r = .82) than on the first (r = −.57). The discriminant function plot indicated that the first function differentiated between hypo- and hyperdivergent participants, while the second function discriminated those from neutral cases. Following the pair of functions, 50.5% of all cases were categorized properly in their vertical group (Table 9). Most incorrect group predictions were found for neutral cases. Hyper- and hypodivergent participants were classified correctly better than by chance.

**Table 9:** Classification of the vertical relationship using condylar width and antero-posterior condylar inclination

Original group	Predicted group			
	neutral	hypodivergent	hyperdivergent	In total
Neutral: n (%)	5 (15.6)	15 (46.9)	12 (37.5)	32 (100)
Hypodivergent: n (%)	2 (5.4)	23 (62.2)	12 (32.4)	37 (100)
Hyperdivergent: n (%)	4 (9.4)	10 (23.8)	28 (66.7)	42 (100)

## Discussion

This study provides an entire overview of the correlation between condylar and craniofacial morphology in all three dimensions – transversal, sagittal and vertical.

Against our expectations, facial asymmetry determined by menton deviation did not mirror side-specific differences in condylar shape or inclination. The only significant side difference was detected for the volumetric ratio C/Mand, which discriminated between the symmetry groups. This indicates that a simple measurement of linear distances and angles is not sufficient to fully identify the complex condylar morphology underlying facial asymmetry. Furthermore, the condyle is only one factor contributing to menton deviation besides the mandibular ramus, body or the joint space [23, 24]. However, the herein displayed volume differences between deviated and non-deviated sides in asymmetric patients are consistent with previous findings [25, 26] and clinical reports of patients with unilateral condylar hyperplasia [27]. Whether in our healthy sample the volumetric difference in condyle morphology caused the menton deviation or vice versa remains to be elucidated.

Interestingly, the vertical and sagittal skeletal relationships independently affected condylar morphology. The antero-posterior condylar inclination was the only factor that discriminated between both the sagittal and the vertical groups. The discriminant analysis revealed the condylar depth, the antero-posterior and medio-lateral condylar inclination and the volumetric ratio C/Mand to correlate significantly with the skeletal class. Based on these four variables, sufficient discrimination between skeletal Class II and III was achieved in most cases, with Class II subjects having smaller, shorter and more inclined condyles compared to Class III subjects. Since Class I individuals demonstrated condylar characteristics in between Class II and III participants, they were rarely identified correctly. This is consistent with Hasebe et al. [15], who reported that condylar height and width increase from Class II to Class I and Class III. Regarding volumetric discrepancies, our results support previous findings by Saccucci et al. [10], who described a tendency for higher condylar volumes in Class III than in Class II patients.

Regarding the vertical relationship, discrimination was best between hypo- and hyperdivergent participants based on condylar width and antero-posterior condylar inclination, consistent with previous research [11, 15]. The volumetric ratio C/Mand, which was higher in hypodivergent than hyperdivergent subjects, was no powerful predictor of vertical relation. This contradicts our expectations based on former reports [10, 28], who found a significantly higher condylar volume in hypodivergent subjects compared to the other groups. However, this reveals the advantages of the applied discriminant analysis, which enabled us to critically evaluate the effect of each variable on the skeletal patterns.

The herein presented strong correlations between condylar characteristics and craniofacial morphology raise the question of whether this interaction is based on functional adaptation or genetics. Previous studies stated that the functional load on the condyles varies according to the individual’s occlusion and skeletal relationships [29, 30]. For example, condyles of patients with hyperdivergent skeletal relationships show a higher prevalence of internal derangement [31] and are supposed to be exposed to increased stress during clenching [32], which might affect condylar shape. Consequently, changes in the craniofacial skeleton induce changes in condylar morphology, as observed in patients undergoing orthognathic surgery [33, 34]. Condylar resorption occurs even in short observational periods of approximately 6 months, especially on the lateral margin of the condyle. This finding supports the hypothesis that the link between condylar and craniofacial morphology is rather based on the adaptability of the condyle than on genetic information.

This study presented objective and reliable criteria to evaluate the condylar shape, avoiding subjective assessments like convex, round, flattened or angled [35]. By a combination of linear, angular and volumetric measurements, we benefited from the full information of the available CBCT/CT data. Participants’ age and gender are known to be associated with condylar morphology [15, 28, 35]. Therefore, the confounders age and gender were compared between the craniofacial groups. While gender was equally distributed between all groups, participants with skeletal Class III were significantly younger compared to individuals with skeletal Class I or II. This is due to the fact that orthodontic therapy of severe Class III patients is often interrupted in adolescence and continued in combination with surgery immediately after growth is completed.

Since the mean age of the whole study population was 27 years, and therefore, the main peak of condylar growth was already past, age effects on condylar characteristics might be negligible in this study. Regarding the generalizability of the results, it has to be considered that we investigated severe Class II and III patients undergoing orthognathic surgery. This study population was chosen purposely to reveal even small differences in condylar characteristics and to overcome the problem of observing only tendencies of correlations between condylar and craniofacial morphology [10, 11]. We assume that the condylar characteristics might be less clear in milder cases. However, it is exactly the difficult orthognathic surgery case that has to be discriminated from the case that can be handled with orthodontic treatment only. Further studies are required to investigate whether the herein identified condylar morphologies are sufficient to predict the skeletal patterns in growing patients.

## Conclusions

In summary, this study demonstrated a clear correlation between extreme skeletal patterns and condylar characteristics in an adult sample. The implementation of a comprehensive 3D analysis of the condylar morphology underpins the versatility of the condyle and is important for understanding the functional dependence between the craniofacial skeleton and the temporomandibular joint. The knowledge of regular condylar variations caused by the skeletal patterns of the patients could be helpful in diagnosis of temporomandibular joint pathologies and could be useful for understanding temporomandibular disorders.

## Data Availability

The datasets used and/or analysed during the current study are available from the corresponding author on reasonable request.
